# Tidal stream to mainstream: mechanical testing of composite tidal stream blades to de-risk operational design life

**DOI:** 10.1007/s40722-022-00223-4

**Published:** 2022-02-28

**Authors:** Conor Glennon, William Finnegan, Nicholas Kaufmann, Patrick Meier, Yadong Jiang, Ralf Starzmann, Jamie Goggins

**Affiliations:** 1 Civil Engineering, School of Engineering, National University of Ireland Galway, Galway, H91 HX31 Ireland; 2 MaREI Centre, Ryan Institute, National University of Ireland Galway, Galway, H91 HX31 Ireland; 3 SCHOTTEL Hydro (Sustainable Marine), Mainzer Straße99, 56322 Spay, Germany

**Keywords:** Tidal energy, Composite blades, Design life, Marine renewable energy, Mechanical testing, Fatigue testing

## Abstract

Tidal energy has seen a surge of interest in recent years with several companies developing technology to harness the power of the world’s oceans where the operational capacity in Europe was over 11 MW in 2020. One such developer is the partnership of SCHOTTEL Hydro (Germany) and Sustainable Marine (UK) who have developed a scalable multi-turbine device equipped with 70 kW turbines and capable of operating in arrays at sites around the world. The technology to harness tidal energy is still at a relatively early stage of development; hence, de-risking of component parts plays a vital role on the road to commercialisation. Despite this, the number of tidal energy blades undergoing test programmes remains small. Two different rotor diameters have been developed for the aforementioned device such that it can be optimised for sites of varying potential. In this paper, a blade from each of the 4.0 m and 6.3 m diameter devices was tested for their responses in natural frequency, static loading and fatigue loading under test standards IEC 62600-3:2020 and DNVGL-ST-0164. Testing saw the survival of a blade in fatigue at a lifetime-equivalent load and the generation of natural frequency, strain and displacement results for both blades. Data generated from the testing as a whole will contribute to the modelling and validation of future tidal blades.

## Introduction

**Fig. 1:**
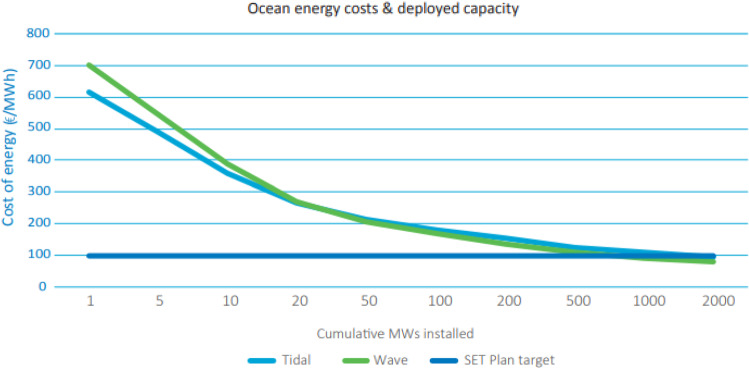
LCOE of tidal and wave energy as capacity ramps up (Cagney 2020)

Europe leads the way in tidal energy globally with a 30 MW installed capacity reached in 2020 (Cagney 2020). In a high growth scenario, Ocean Energy Europe (OEE) (Cagney 2020) foresees 2.9 GW of installed ocean energy capacity worldwide by 2030, with 2.6 GW of this in European waters. Furthermore, with 45% of Europe’s citizen’s living in coastal regions, OEE predicts that ocean energy can meet 10% of EU electricity demands by 2050. Installations at this scale will have the associated benefit of reducing the levelised cost of tidal energy (LCOE) towards a target of €100/MWh (Fig. 1). LCOE reductions of this order will make ocean energy a viable compatriot to offshore wind, which has benefitted from the efficiencies of large-scale deployment.

Extracting energy from tidal flows requires a robust mechanical system to convert the kinetic energy of the water into rotational torque and ultimately electrical power. Turbine blades must be capable of absorbing the high-power output of these machines for a typical 20-year design life in very harsh conditions—submarine turbine blades contend with a lifetime of saltwater immersion and sometimes collisions with floating debris.

Design and testing of such turbine blades is a key factor in the success of any device. Engineers must balance the opposing demands of hydrodynamic and structural efficiency to produce an effective turbine blade. In order to improve the design of tidal turbine blades, a number of advanced computational design methodologies have been developed (Fagan et al. 2016; Finnegan et al. 2020; Jiang et al. 2019; Kennedy et al. 2018), which are based on finite-element analysis and computational fluid dynamics. Large-scale structural testing of tidal turbine blades and components has taken place in recent years to de-risk the structural integrity of blades, prior to deployment. In 2017, fatigue testing was conducted for a 3/8th scale blade component and rotor subsection for the OpenHydro prototype tidal turbine (de la Torre et al. 2018). In 2020, a static and cyclical testing programme was completed on a helical foil for the ORPC device (Meier et al. 2020) and an advanced structural testing programme was completed on a full-scale blade for the Orbital Marine Power tidal turbine, where fatigue cycles for the equivalent of 20 years operation were completed on the blade. During the static testing of the latter, the largest load ever reported on a tidal energy turbine blade was applied, which was in excess of 1000 kN (Garanovic 2020).

**Fig. 2:**
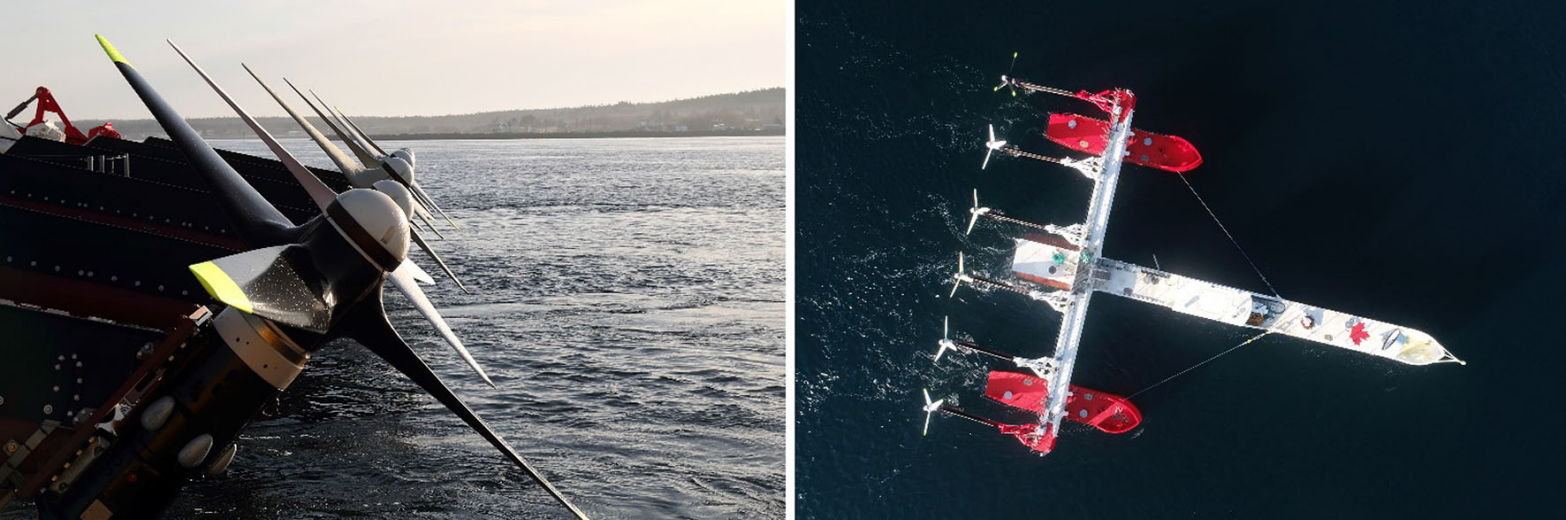
Sustainable Marine Energy’s device testing on-site (Sustainable Marine 2021)

Marine renewable energy as a technology is still in its early stages of maturity in comparison with other renewables. This is clearly visible in the range of technologies currently being pursued, mainly in Europe. Orbital Marine Power based in the UK recently deployed a twin rotor in-stream device at the EMEC site in Scotland (Watt 2021). ORPC based in Maine USA ae developing their ‘RivGen’ device to exploit run-of-river potential at sites in remote parts of Alaska and elsewhere in the USA. Recently the RivGen device passed 7 million operational cycles including a springtime contending with frazil ice as it melted and flowed downstream (Kist 2020). SCHOTTEL Hydro (Germany) together with Sustainable Marine Energy (UK) have developed their current SCHOTTEL Instream Turbines (SIT), on the surface of the floating inshore platform PLAT-I. The first generation system has undergone sea testing using 4 m rotors between November 2017 and June 2018 in the UK and was deployed in Nova Scotia (Canada) from September 2018 to February 2021 with 6.3 m rotors (Starzmann et al. 2019). PLAT-I 6.40 is Sustainable Marine’s second-generation floating platform. Figure 2 shows the platform at Grand Passage, Nova Scotia. It is a three-hulled tidal energy platform that hosts six SIT 250 turbines. The platform self-aligns to incoming flow via a mooring turret, which is connected to a geostationary mooring spread. The turbines are suspended from the cross-deck, via lifting support structures. During normal operation, the turbines are in the down configuration, but they can be lifted clear of the water for maintenance. Figure 2 shows the platform in parked mode, with turbines up in Grand Passage. The SIT 250 is a horizontal axis instream turbine. The first generation SIT 250 was presented by SCHOTTEL in 2012 (Jeffcoate et al. 2015). It is designed as a modular turbine system utilizing one drivetrain for two rotor diameters, 4 m and 6.3 m, which can be selected based upon the varying velocity frequency distributions of different deployment sites. The larger rotor diameter is suited to lower flow speed sites, whereas the smaller rotor diameter is suited for higher resource sites. The SIT 250 drive train is rated at the mechanical shaft, so rated power and grid-ready electrical power are *P*_rated_ = 85 kW and *P*_el_ = 70 kW, respectively. Both three-bladed rotor versions are manufactured from composite material to provide passive-adaptive pitch behaviour. The blades are fitted onto a standardized hub. A slow-speed shaft, planetary gearbox and asynchronous generator complete the drive train; the system is cooled by ambient water. The full-scale turbine was extensively tested on Sustainable Marine’s first-generation surface floating PLAT-I platform and showed good agreement with semi-empirical power and thrust predictions using BEM (Starzmann et al. 2018; Lake et al. 2021).

This paper focuses on the mechanical testing of two turbine blades—one blade from each of the 4.0 m and 6.3 m diameter SIT250 devices—that were tested at the Large Structures Laboratory at the National University of Ireland Galway (NUI Galway), Ireland. Testing involved initial static testing, where the blades were required to satisfactorily hold a load for a period of at least 10 s, to ensure they had the required strength. Cyclic and fatigue testing was conducted to contribute to knowledge on turbine blade life expectancy and cumulative damage. Finally, natural frequency and static testing were carried out at various time points to develop a relationship between changes to blade natural frequency and residual blade strength, i.e. to assess damage over the design life of the blade.

The successful completion of the accelerated 20-year fatigue testing on the SCHOTTEL blade will be one of the first times that a tidal blade has been proven over its full design life in fatigue.

## Materials and methods

### Aims and objectives

Component parts of tidal energy devices must be rigorously tested to validate their design and manufacturing processes. The aim of this study was to qualify two composite tidal turbine blade designs for a 20-year design life through a suite of mechanical tests. Design, material selection and manufacturing of the candidate blades was carried out by SCHOTTEL Hydro (Germany) and Sustainable Marine Energy (UK) and produced two separate blade designs; the first, a ~ 3 m length blade for a 6.3 m diameter rotor (hereafter referred to as the D63 blade) and the second, a ~ 2 m length blade for a 4.0 m diameter rotor (referred to as the D40 blade). Both blades are intended for a 70 kW device, with the longer blades optimised for lower potential sites.

The study carried the following objectives from the testing to prove blade life:

- Validation of the structural design methodology of passive-adaptive tidal turbine blades with respect to stresses, deflection, and blade twist during static testing.
- Validation of design assumptions with respect to mode shapes and natural frequencies.
- Prove the integrity of the blade design for ultimate loads in accordance with International Electromechanical Commission IEC TS62600-3 (IEC 2020) and DNVGL-ST0164 (DNV GL 2015) standards.
- Prove the integrity of the blade during accelerated lifetime/dynamic testing in accordance with the aforementioned standards.
- Analyse the structural behaviour of the novel attachment system solution (clamped blade/hub connection) under static and cyclic load.
- Feed results into review and next-generation blade designs/manufacturing methods.

### Blade description and materials

**Fig. 3:**
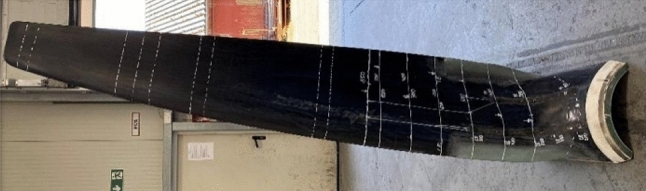
SCHOTTEL SIT250 blade

Two tidal blades are considered in this study; the 2 m length D40 blade and the 3 m length D63. Both are resin-infused carbon fibre blades. In each case, the blade forms part of a single-rotor, three-blade device with a swept diameter of 6.3 m and 4 m, both producing a rated 70 kW electrical output from low flow and high flow sites, respectively. Special attention was given to the hydraulic design of the turbine blades. A novel multi-objective optimizing scheme, described by Kaufmann (2019), has been developed targeting the best compromise between maximum power output, minimum thrust load and shallowest immersion depth for operation without cavitation. To support the passive-adaptive pitching, the stacking line of the blade has been designed to provide a sufficient positive moment around the radial axis. The blade-hub connection design is achieved using a novel clamping mechanism; blade fibres run continuously from the blade tip radially towards the blade root. When approaching the blade root, the continuous fibres turn gradually through 90° from a radial (spanwise) direction to axial direction. The axially oriented fibres mate against the cylindrical rotor hub, where they are clamped top and bottom using a tapered locking ring fastened in place with bolts. This hub connection design is novel in the industry, as it does not require through holes drilled in the composite or adhesive joints. Figure 3 shows the finished blade.

### Blade manufacture

In both cases, the test blades were manufactured in two halves by infusing carbon fibre fabric with an epoxy resin through a process called vacuum-assisted resin transfer moulding (VARTM). VARTM is widely using in the composites manufacturing industry and involves laying up dry carbon fibre fabric into a mould. The mould/fabric assembly is then vacuum bagged and placed under vacuum pressure for a period to evacuate the air. Resin is infused into the fibre by connecting a vacuum line from the layup to a resin mix stored in an open container. The pressure differential between the resin mix open to the atmosphere and the evacuated layup pushes the resin into the layup. The evacuated layup ensures inter- and intra-tow wet out of the carbon fibre with minimal voiding or air bubbles included. Pressure and suction blade halves were produced in this way before being bonded with an epoxy adhesive along their neutral axis to produce a blade.

Blade shells were manufactured from a layup complex of unidirectional (UD) and bi-axial (BX) carbon fibre fabric infused with an epoxy resin. The layup of UD and BX fabric was optimised to suit the expected loading conditions with the UD reacting the main bending loads and the BX taking the shear and torsional loads. A shear web was pre-fabricated offline using quad-axial carbon fibre fabric and included in the final glue between the pressure and suction shells. Blades were then finished to a smoother hydrodynamic shape by abrasion of positive areas or glue squeeze-out and the filling of negative areas, where necessary.

### Test methodology

An extensive programme of mechanical testing was developed and comprised of:

1. Determination of the test loads.
2. Natural frequency testing prior to and post mechanical testing.
3. Static testing up to and beyond the 20-year design load.
4. Fully reversed fatigue testing at 20-year design load.
5. Residual strength testing, which includes both natural frequency tests and static tests.

Each of these phases of the project is described in the following sub-sections.

#### Dynamic load simulation

To derive the test loads, dynamic load simulations of the SIT250 turbine were performed using the TIDAL BLADED® software by DNVGL-Garrad Hassan. TIDAL BLADED® is the industry-standard software for tidal stream turbine design calculations and has been validated against full-scale measured data (Parkinson and Collier 2016). The hydrodynamic model of the software is based on the BEM theory. Through the coupling to a structural model, the load-related deformation of the blades can be resolved. The full model includes, besides the hydrodynamic and structural properties, the turbine controller and dynamic effect due to 3D turbulence, waves and the wake of the structure upstream of the turbine.

The load cases are based on the DNVGL-ST-0164 2015 standard. The three key limit states assessed are the Ultimate Limit State (ULS), Accidental Limit State (ALS) and Fatigue Limit State (FLS). The ULS design represents extreme load events during all expected scenarios of the turbines system’s operating life. The FLS cases capture all contributions to fatigue damage within the targeted design life. The turbine’s relevant ALS case, a grid loss, is considered within the ULS states. The turbine operational states analysed are power production, normal stop and idling (freewheeling). The basis is data gathered from a measurement campaign that deployed an Acoustic Doppler Current Profiler at the installation site in the Minas Passage of the Bay of Fundy, Canada. For each load case, 10 min samples are simulated. In cases of a stop (normal or forced), the simulation time is adapted accordingly.

For each FLS case, the annual occurrence frequency is derived from the field data and a subsequently performed harmonic analysis. Thus, the occurrence frequency of each FLS case within the targeted operating years can be determined. The Rainflow Counting Method analyses the load-time history for each simulation to identify the number, range and mean of load cycles. The method is described in “Standard Practices for Cycle Counting in Fatigue Analysis” ASTM E 1049-85 (ASTM 2011). The output from the rainflow cycle counting analysis consists of the two-dimensional distribution of the number of cycles binned on the means and ranges of the cycles, often referred to as *Markov matrix* or *Rainflow matrix*. Thus, the structural damage caused can be calculated considering the number of cycles, mean stress and range. Subsequently, the total damage can be determined, e.g. by applying the Pilgrim-Miner rule. The test programs are optimized to cause the equivalent damage as the resulting fatigue loads over the target service life of twenty years.

#### Natural frequency testing

Natural frequency tests were conducted to characterize the dynamic properties of the turbine blade. Testing methodology was carried out in line with DNVGL-ST0164 and IEC 62600:3 standards. Acceleration response of individual points on the blade surface was measured either using accelerometers or a laser scanning vibrometer under free damped vibration (refer to Sect. 2.4.6.3 for further details). In each test, the blade was struck on the tip pressure side quickly and smoothly by hand to impart a transient impact and induce free damped vibration. The measured acceleration responses were transferred to a response spectrum by fast Fourier transform (FFT) and the first and second-order natural frequencies were calculated from the response spectrum. The half-power bandwidth method was used to estimate the damping ratio for the first flapwise mode from the response spectrum. It should be noted that damping in the second-order flapwise mode shape is not required as per DNVGL-ST0164. Hydrodynamic damping experienced by submerged rotor blades in the water will be high compared to aerodynamic damping experienced by wind turbine blades, so the importance of structural damping for tidal turbine blades is of less importance than wind turbine blades, as damping of vibrations of the blades during operation is caused by the relatively high viscosity of the surrounding fluid. Thus, for verification of the hydrodynamic damping and added mass, it is expected that relevant measurements are carried out during prototype deployment (DNVGL-ST0164), which is outside the scope of this paper.

**A. D40 blade**

Testing was conducted to determine the natural frequency of the blade at different points in time during the fatigue test programme. Accelerometers recorded accelerations in the vertical axis during testing. In the case of the D40 blade, testing was conducted without the load introduction clamp installed. Three measurements were conducted for each test run and the average response was used to minimise the effect of noise.

**B. D63 blade**

**Fig. 4:**
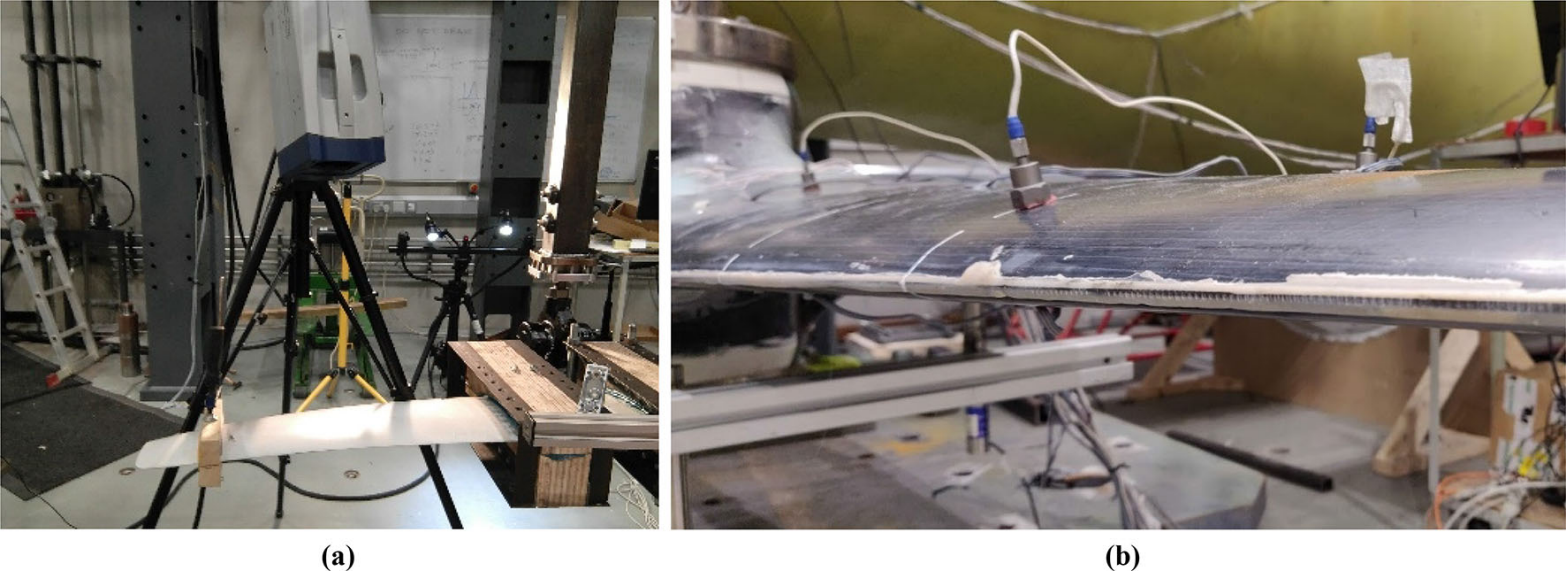
Setups for natural frequency testing showing **a** DIC setup for the D63 blade and **b** single-axis accelerometers installed on the D40 blade

During testing of the D63 blade, a PSV-500 Laser Scanning Vibrometer (shown in Fig. 4a) was utilised to scan the acceleration response of each individual point on the blade surface under free damped vibration. There were four Laser Doppler Anemometry (LDA) investigations performed at different test stages, with details listed in Sect. 3.1. For each LDA measurement, several points were scanned to make sure that all the natural frequencies were captured in the adopted measuring range (0–100 Hz). It should be noted that the first three LDAs were performed with two clamps glued to the test blade. This would influence the blade response and test results accuracy and is discussed in the results section.

#### Static testing

Load cells were either zeroed (tare value) or the starting static load was noted at the start of each test programme. Since absolute values of displacement and load are used, this tare reading included the weight of the pivot acting on the load cell. Testing was conducted to determine the load–deflection relation and verify that the test setup worked as intended. For each test run, the load was ramped to the target test load, held for at least 10 s in compliance with the DNVGL-ST0164 standard and subsequently released to the neutral position.

**A. D40 blade**

During multiple test runs, the maximum load was increased stepwise until the target load of the dynamic tests of + 6.9 kN and − 6.9 kN was reached. This procedure was performed for loads both in the suction direction (top surface of blade as mounted, yielding negative loads and actuator displacement) and the pressure direction (bottoms surface of blade as mounted, yielding positive loads and displacements) loads. The load actuator was controlled manually during these tests. Test runs are summarised in Table 1. Deflections during static testing were noted to inform the fatigue testing.

**Table 1:** D40 static test loading details

Test run	Load direction	Test load [kN]	Holding time [s]	Notes
1	Suction	− 2.5	30	Initial static testing, increasing to − 6.9 kN max load
2				
3				
4		− 5.0		
5				
6				
7		− 6.9		Max tensile static test load
8				
9				
10				Repeat of the three 6.9 kN tensile static tests
11				
12				
13	Pressure	2.5	30	Initial static testing, building up to + 6.9 kN max load
14				
15				
16		5.0		
17				
18				
19		6.9		Max compressive static test load
20				
21				
22	Suction	− 10.0	30	Additional static loading post fatigue
23	pressure	10.0		

**B. D63 blade**

Static loading up to a maximum of + 20 kN was applied during testing. Static loads were only applied in the pressure (+ ve loads) direction. Table 2 summarises the static loading runs.

**Table 2:** D63 static loading test details

Test run	Load direction	Test load [kN]	Holding time [s]
1	Pressure	5	10
2		10	10
3		15	10
4		20	10

#### Fatigue testing

The main body of fatigue testing was conducted on the D40 blade and imparted the equivalent damage of 20 years of operation to the blade. A more limited cyclic loading programme was carried out on the D63 blade. Blade and installation fixtures were periodically inspected during testing for signs of movement or damage. Test frequency was governed by the ability of the hydraulic system and load control software to achieve the necessary displacement at the blade, while also seeking to avoid localised heating of mating surfaces in the blade clamp/mounting region.

The results of load simulation on the D40 blade were used to calculate a single load collective of 150,000 sinusoidal cycles at fully reversed loading of ± 6.9 kN, which was expected to cause an equivalent damage to the blade material under 20 years of operation. Cyclic testing of the D63 blade was carried out to a target load of ± 14.0 kN fully reversed loading. Table 3 summarises both fatigue programmes.

**Table 3:** Fatigue test definition for D40 blade

Blade	Target max. test load [kN]	Min. test load [kN]	Load ratio [–]	Number of cycles	Cycle type	Frequency [Hz]	Notes
D40	+ 6.9	− 6.9	− 1	150,000	Sinusoidal	0.30	Main phase
D63	+ 14.0	− 14.0	− 1	16,000	Sinusoidal	0.1	Main phase

#### Load introduction, application and control

**Fig. 5:**
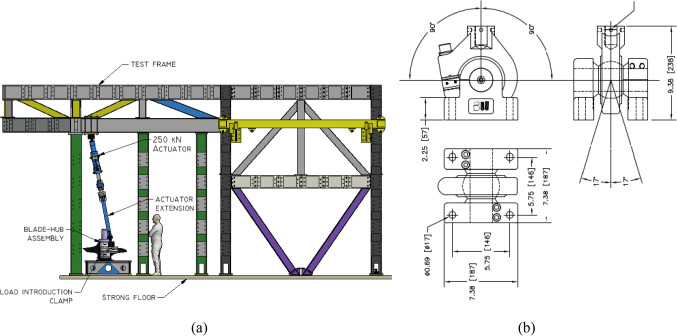
**a** Actuator inclined 11° to the vertical, D40 blade projecting out of page, **b** swivel joint (model Shore Western 983) for load introduction

A servo-hydraulic actuator with a capacity of 250 kN and max stroke of 250 mm was used for static and fatigue testing for both the D40 and D63 blades. The actuator line of action was inclined at an angle to the vertical to induce a combined edgewise and flapwise loadings (see Fig. 5 which shows the D40 setup). The inclination was 11° for the D40 and 9.5° for the D63. Load introduction in both cases was achieved using profiled wooden clamps installed at the radial distances from the root as summarised in Table 4. Load was transferred between the actuator and the clamps in both cases using a Shore Western swivel joint (Model 983) as shown in Fig. 5b. The Shore Western joint ensured load was transferred to the clamp as a zero-moment point-load throughout its full range of motion.

**Table 4:** Load introduction position

Blade	Position no	Radial position [mm]	Blade length [mm]
D40	1 of 1	1600	2000
D63	1 of 2	2047	3100
	2 of 2	2362	

**Fig. 6:**
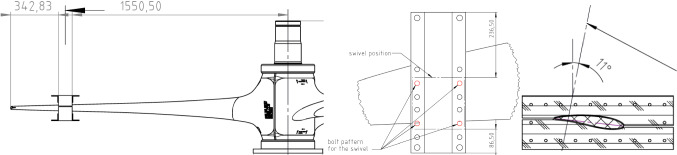
D40 blade positioning of load introduction clamp

**Fig. 7:**
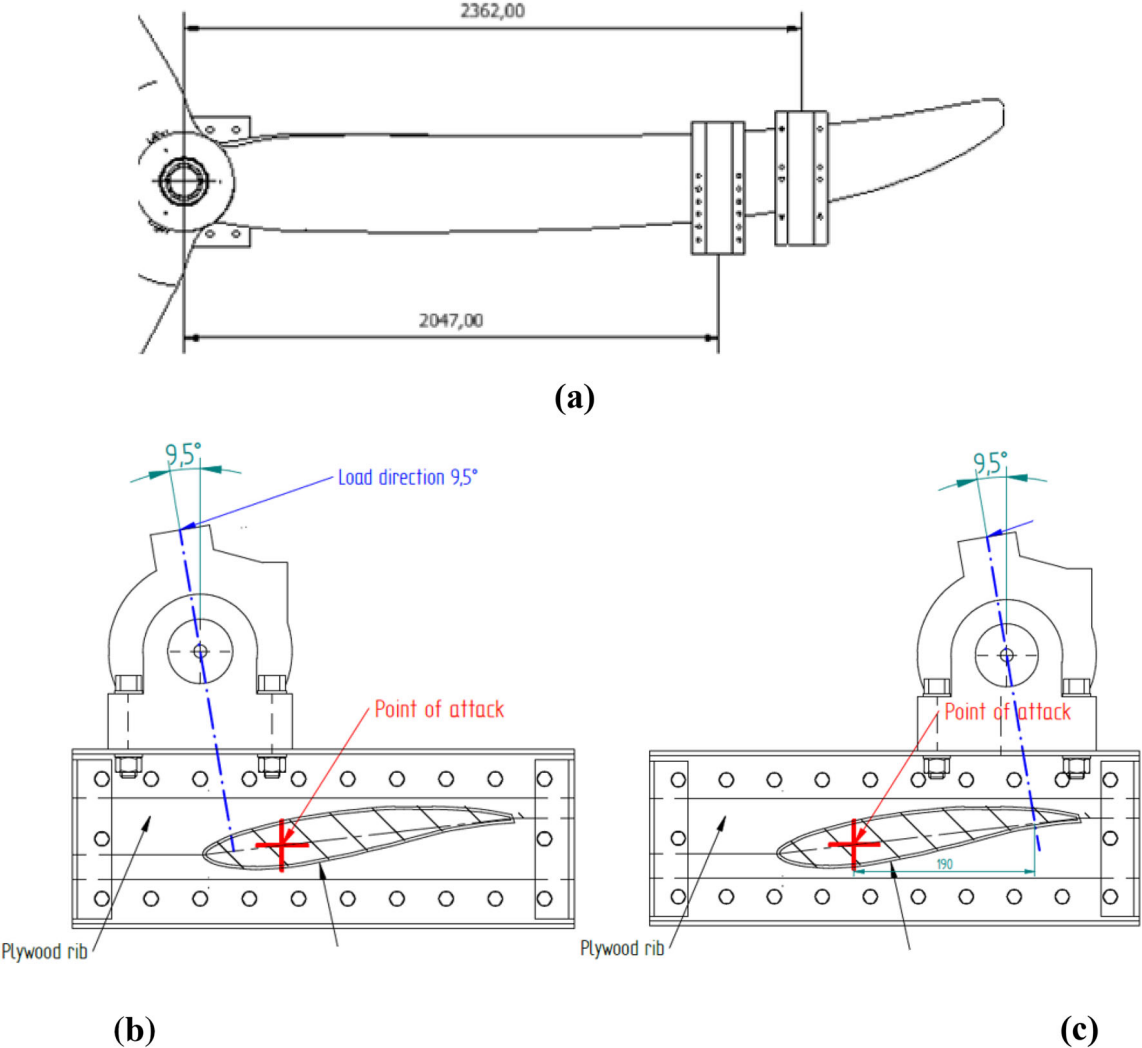
D63 load introduction clamp positioning, **a** plan view, **b** cross-section at 2047 mm location, **c** cross-section at 2362 mm location

The load actuator was controlled using Cubus® software and tests were performed under displacement control, where the digital controller and software ensure the actuators automatically applied constant displacement amplitudes to the sample. Displacement control is useful in safely limiting the displacement amplitude of a weakening structure as testing progresses. Required displacements were determined experimentally from static testing as per Sect. 2.4.3. For the fatigue test, the loading profile was sinusoidal with a period determined by the ability of the actuator and software to achieve the desired displacements. Applying a sinusoidal loading profile results in minimal perturbations and enhanced control of the load as it shifts direction. For the static test, the test duration is governed by the standard DNVGL-ST0164 (Figs. 6, 7).

#### Instrumentation

The test blades were instrumented extensively prior to testing to gather as much data as possible from the test programme. Instrumentation used during testing included:

- Two displacement sensor types:
  o Linear Variable Displacement Transducers (LVDTs) for small displacements < 25 mm.
  o Draw-wire displacement sensors (stringpots) for locations where larger displacements were expected.
- Electrical resistance strain gauges applied to the surface of the blade.
- Load cells at the location of load application to the blade recorded the instantaneous load at a pre-defined frequency.
- Accelerometers for natural frequency tests.

Instrumentation is described below in turn for the D40 and D63 blades.

##### Strain sensors

Electrical resistance strain gauges manufactured by the Tokyo Measuring Instruments Lab were used and were of two types, linear and rosette. Linear strain gauges were used on locations along the blade length where the strain was primarily linear, while rosette gauges were used near the blade root to record strain along three axes. FEM indicated the areas of high strain which guided the sensor placement. Details of the strain gauges are summarised in Table 5. Strain gauges were named using the following convention, Blade identifier—Suction/Pressure side—Chordwise position bias—Spanwise position [mm]—Linear or rosette type. For example, the strain gauge identified as 40-SS-TE-300-ROS belongs to the D40 blade—Suction Side—Trailing edge biased chordwise placement—300 mm radial station—rosette type.

**Table 5:** Strain gauge details

Strain gauge type	Orientation	Resistance	Strain limit	Manufacturer	Model no
Linear	0°	120 ± 0.5 Ω	5%	Tokyo Measuring Instruments Lab	FLAB-6-11-3LJCT-F
Rosette	0°/ + 45°/− 45°				FRAB-6-11-3LJBT-F

**A. D40 blade strain sensor locations**

**Fig. 8:**
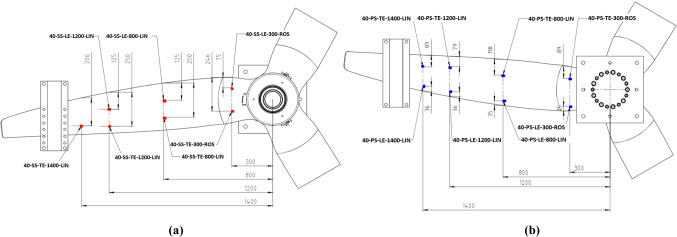
D40 blade strain gauge locations

Strain gauges were installed at 15 positions distributed on the suction side (SS) and pressure side (PS) of the blade. 11 no. linear and four no. rosette strain gauges were applied to the blade as per the strain gauge map in Fig. 8b. The four rosette strain gauges were installed at the blade root, two each on the suction surface, Fig. 8a, and pressure surface, Fig. 8b. Rosette strain gauges were of the 0°/ + 45°/− 45° type with the 0° axis aligned with the spanwise direction.

**B. D63 blade strain sensor locations**

**Fig. 9:**
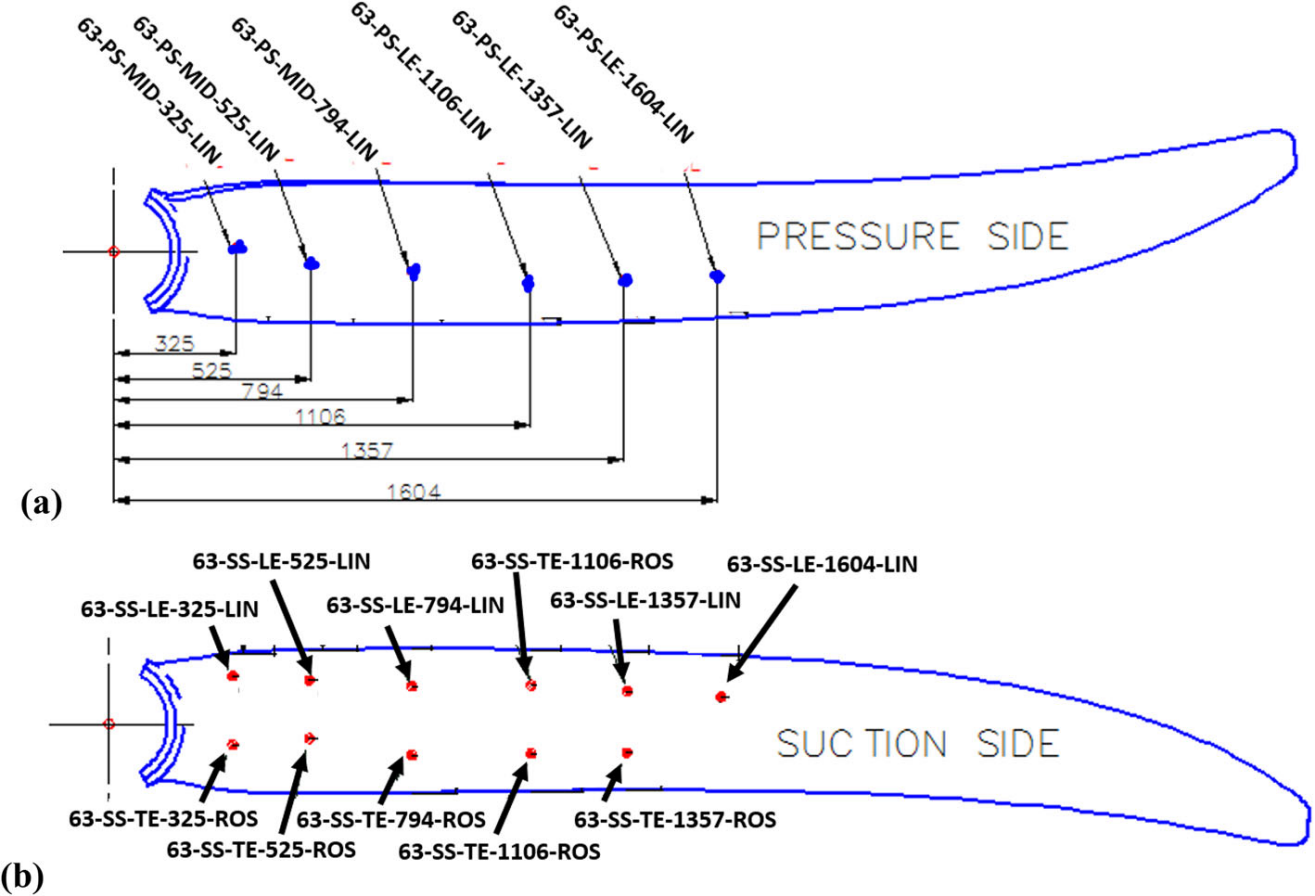
D63 blade strain gauge locations

The D63 blade used 17 no. strain gauges, which were 12 no. linear and 5 no. rosette gauges. Figure 9 shows the installation locations using the same naming convention as the D40 blade.

##### Displacement monitoring

Instrumentation to monitor displacement was installed at several locations on both the blade and test fixture. Sensors on the blade measured blade flexure and blade twist, while instrumentation on the fixture was used to evaluate the suitability of the test fixture and confirm the validity of the boundary conditions.

To monitor the foil’s deflection during the test, Linear Variable Displacement Transducers (LVDTs) and string pots were used. Two different LVDT models were used depending on expected displacement:

- RDP Electronics *D6/05000A* with a range of ± 5 mm and sensitivity 700 mV/V
  o TX, TY on the D40 blade.
- RDP Electronics *ACW1000A* and *ACT1000A* both with a range of ± 25 mm and sensitivity 900 mV/V
  o LVDT_L10, LVDT_T10 on the D40 blade.
  o LVDTLE, LVDTTE on the D63 blade.

Two models of draw-wire displacement sensors (string pots) were used:

- WDS electronics *WDS-500-P60* with a range of + 500 mm and linearity of ± 0.5 mm
  o POT_L16, POT_T16, POT_TIP on the D40 blade.
  o SP500LE, SP500TE on the D63 blade.
- WDS electronics *WDS-1000-P60* with a range of + 1000 mm and linearity of ± 0.2 mm
  o SP1000LE, SP1000TE on the D63 blade

**A. D40 blade monitoring locations**

**Fig. 10:**
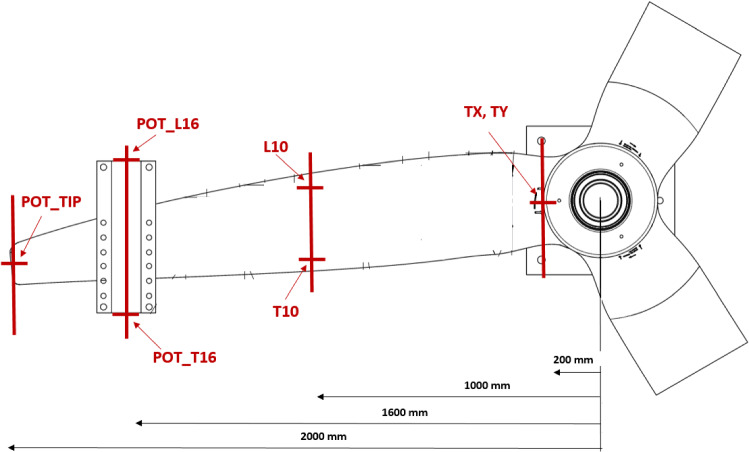
LVDT and string pot installation locations on the D40 blade

Displacement of the D40 test setup was measured at seven locations as described in Table 6 and Fig. 10. Deflection at the leading and trailing edges was measured to determine the torsion about the foil axis. Only mid-chord deflection was measured at the tip, as it was not practical to install leading and trailing edge displacement sensors.

**Table 6:** Displacement sensor locations on D40 blade

Sensor name	Sensor type	Radial location [mm]	Orientation	Subject	Chordwise position	Range
TY	LVDT	200	Vertical	Hub mount	n/a	± 5 mm
TX	LVDT	200	Horizontal			± 5 mm
LVDT_L10	LVDT	1000	Vertical	Blade pressure side at *r *= 1000 mm	LE	± 25 mm
LVDT_T10	LVDT	1000	Vertical		TE	± 25 mm
POT_L16	Stringpot	1600	Vertical	Blade pressure side at *r* = 1600 mm	LE	± 500 mm
POT_T16	Stringpot	1600	Vertical		TE	± 500 mm
POT_TIP	Stringpot	2000	Vertical	Blade tip	MID	± 500 mm

**B. D63 blade monitoring locations**

**Fig. 11:**
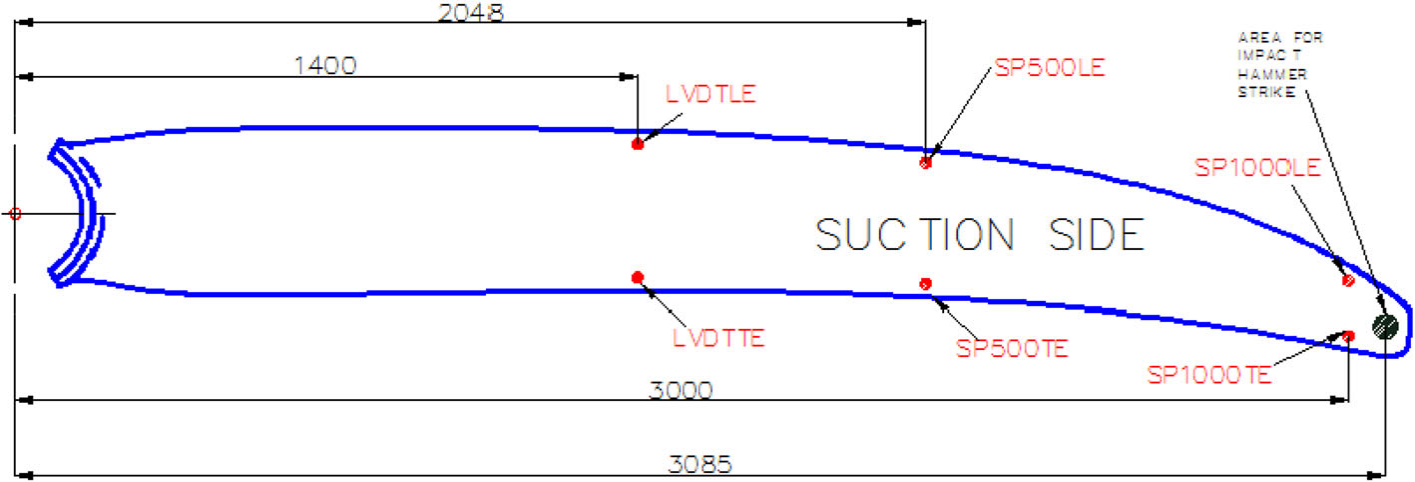
D63 blade LVDT and stringpot locations

To monitor blade deflection, LVDTs of the RDP *ACW1000A* type named LVDT LE and LVDT TE were placed in two positions in the initial setup attached to an extruded aluminium frame attached to the floor, according to the map shown in Fig. 11. Additional LVDTs of the RDP Electronics *D6/05000A* type were later added to monitor apparent movement in the hub connection. For those locations where expected deflections were outside of the range of the available LVDT stroke, string pots were attached. The *WDS-500-P60* string pots (range 500 mm) were attached to the 2048 mm offset blade clamp, and the *WDS-1000-P60* string pots with increased range were attached near the tip to a dedicated blade clamp.

Additionally, the D63 blade also benefitted from digital image correlation (DIC) which recorded the movement of the blade surface using datums marked on the blade surface for tracking movement during static and cyclic tests. The DIC system used in the Heavy Structures Laboratory at NUI Galway is the ARAMIS 3D 5 M. The unit consists of an ARAMIS stereo 5 M sensor head, including two cameras mounted on a stable camera frame with laser pointer and dual-LED illumination. The system is also equipped with a high-end portable laptop and sensor controller, supporting synchronised analogue data acquisition and advanced triggering options. The lenses used in the system are suitable for measuring areas from 130 × 110 to 1050 × 880 mm^2^. Two methods for capturing displacements and strains are available using the DIC; measurements of a stochastic painted pattern and tracking reference point markers. For the D63 blade, the reference point marker method was used extensively. The reference point markers were created by first applying a base layer of black to the surface of the test object, then applying a pattern of white dots. The markers were manually applied using UNI POSCA PC-1MR 0.7 mm pens.

##### Acceleration sensors

Acceleration sensors were installed at positions along the length and chord of the blade. Such positioning was necessary to capture the first and second flapwise natural frequencies and the first torsional natural frequency. Recording of acceleration for each blade was done by one of two methods according to the blade type. The use of different methodologies for the collection of acceleration data was a decision based on the paper by Jiang et al. (2020), which concluded that both methodologies have a high level of accuracy for a range of vibration amplitudes. The decision to use accelerometers in the D40 testing was thus largely based on the fact that the accelerometers would use the same data acquisition system that was already configured for the blade to gather displacement and load information.

**A. D40 blade accelerometer locations**

Surface-mounted accelerometers to record blade displacement as a function of time. Accelerometers were of the lightweight piezoelectric type, manufactured by Endevco. The specific accelerometer used was the 752A12 with a range of 1–8000 Hz and a sensitivity of 100 mV/g.

**Fig. 12:**
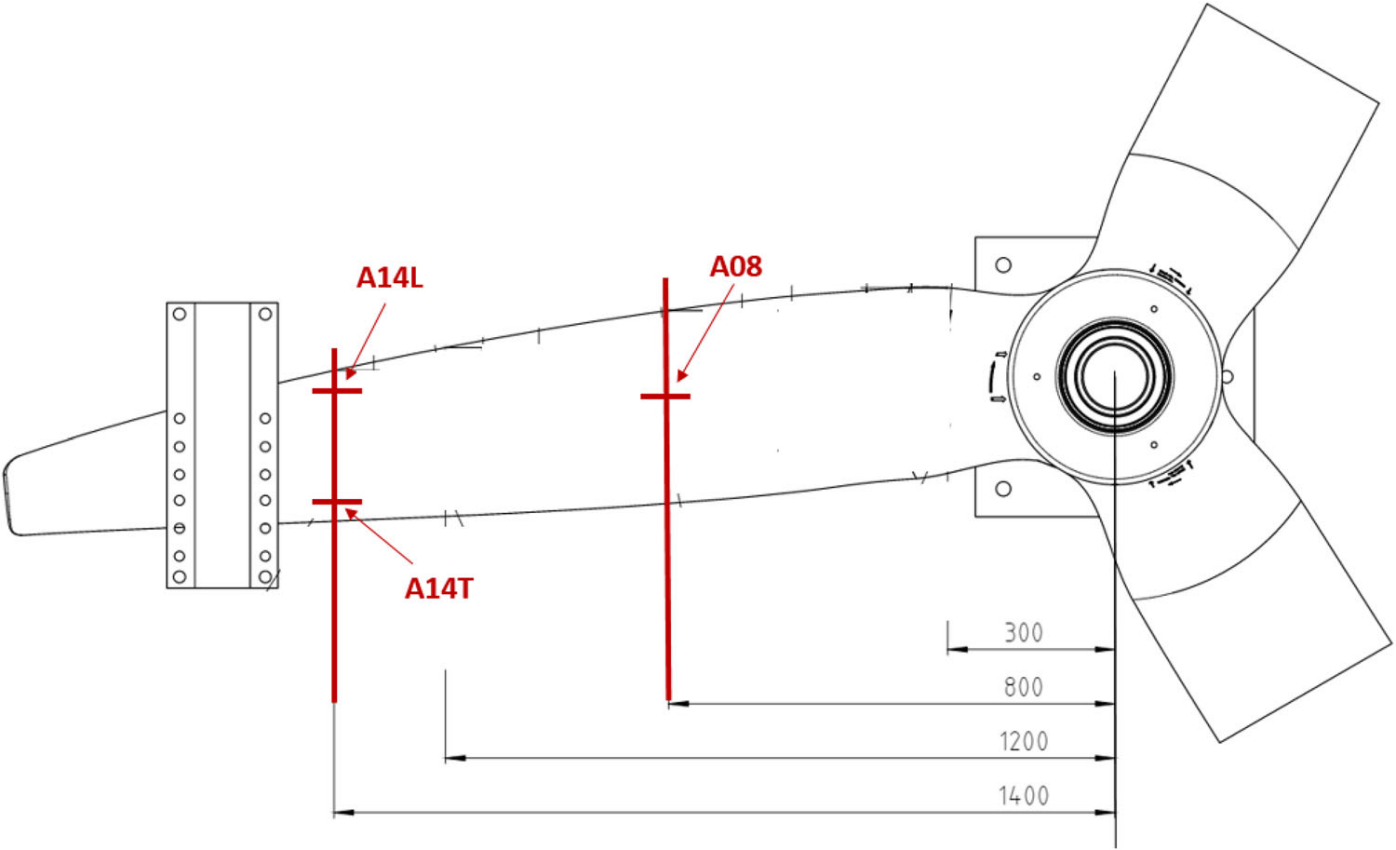
Accelerometers mounted on the blade pressure surface

Three single-axis piezo-electric accelerometers, model *752A12,* were installed on the blade suction surface at *r* = 800 mm mid-chord, *r* = 1400 mm trailing edge and *r* = 1400 mm leading edge, as shown in Fig. 12. The sensors were fixed to the blade using hot wax to hold them in place. Accelerometers recorded local acceleration as a function of time, which could then be processed into frequencies using Fast Fourier Transform methods.

**B. D63 blade acceleration monitoring**

Acceleration date for natural frequency determination was generated using a laser vibrometer system. The PSV-500 Laser Scanning Vibrometer was utilised to scan the acceleration response of each individual point on the blade surface under free damped vibration. The laser vibrometer works based on the Doppler effect to produce a Laser Doppler Anemometry (LDA) analysis. The measured acceleration responses were transferred to the response spectrum by fast Fourier transform (FFT). The natural frequencies were calculated from the response spectrum.

## Results

### Natural frequency testing

**A. D40 blade**

Using the accelerometers installed on the blade to record the response, the tip of the blade was struck in the flapwise direction to excite it into free vibration. Each test was repeated three times. The average was taken to estimate the natural frequencies, natural period, damping and mode shapes of the blade, which are summarised in Table 7.

**Table 7:** D40 blade natural frequency testing results

	Mode no	Frequency [Hz] (CoV)	Period [s]	Damping [%]	Notes
Pre-testing	1st flapwise	34.3 (0.001)	0.029	0.98	Load introduction clamp not installed
	2nd flapwise	44.0 (0.003)	0.023	Not applicable as per DNVGL-ST0164	
	1st torsional	132.2 (0.001)	0.008		
Post-testing	1st flapwise	33.4 (0.001)	0.030	1.06	
	2nd flapwise	45.1 (0.006)	0.022	Not applicable as per DNVGL-ST0164	
	1st torsional	132.2 (0.002)	0.008		

**Fig. 13:**
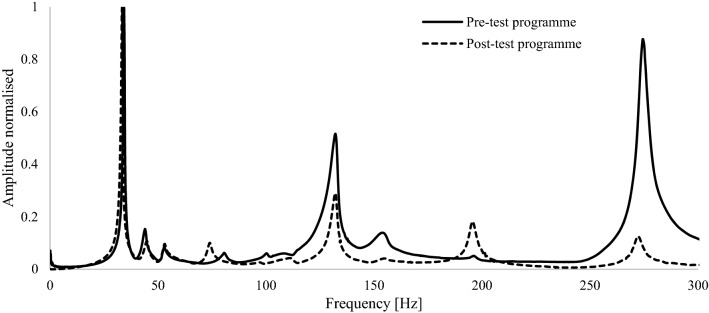
Co-plot of natural frequency test results pre-testing and post static and 150,000 cycle fatigue testing

Figure 13 plots the results of the natural frequency testing. ‘Pre-test programme’ natural frequency testing was carried out prior to any static or fatigue testing. ‘Post-test programme’ testing was carried out  after the normal static and fatigue programme, not including any additional testing.

**B. D63 blade**

The natural frequency response of the D63 was recorded in a different manner to the D40 blade and used light-based digital recording of the blade’s free vibration response to a disturbance, see Sect. 2.4.6.3.

**Fig. 14:**
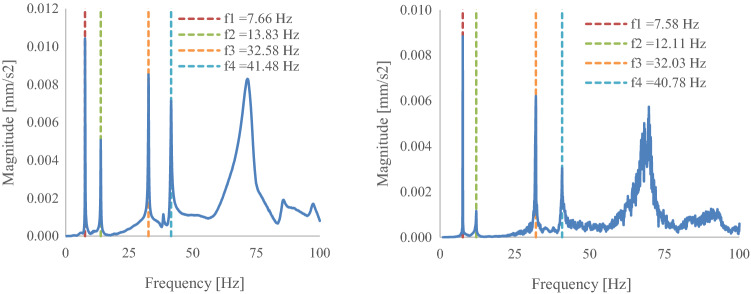
Average acceleration response spectrum at blade root (with clamps)—pre-fatigue (**a**), post-fatigue (**b**)

**Fig. 15:**
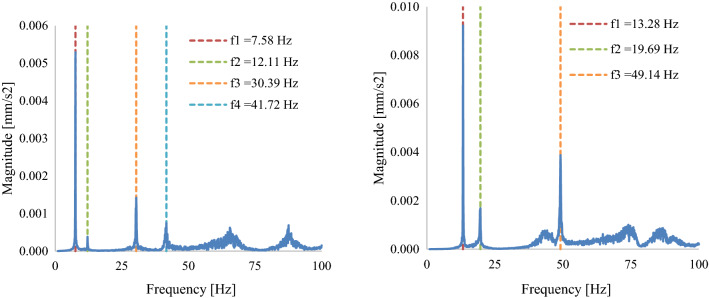
Average acceleration response spectrum at blade root post-testing, with clamps (**a**) and without clamps (**b**)

Figures 14 and 15 show the average acceleration response spectrum of the four LDA (Laser Doppler Anemometry) runs. From initial testing, it was noted that the root of the blade is sensitive to all the modes in the frequency range 0–100 Hz, while the blade tip is only sensitive to the 1st and 3rd modes. Thus, for LDAs 2, 3, 4 only the blade root was scanned. Table 8 summarises the natural frequencies of the first two modes of the blade as required by the DNVGL-ST0164 standard (DNV GL 2015). It should be noted that the first three LDAs were carried out under the condition that two clamps were attached to the blade, which introduced additional mass to the blade. Thus, the natural frequency values measured from LDA-3 are significantly different than the values of LDA-4 Post testing. As only one run of the laser scanning vibrometer was done for each test, there is no coefficient of variation applicable to the data.

**Table 8:** D63 blade natural frequency testing results

LDA		Mode no	Frequency [Hz]	Period [s]	Damping [%]	Notes
1	Pre-fatigue testing	1st flapwise	7.66	0.131	0.1	Load introduction clamp installed
		2nd flapwise	13.83	0.075	Not applicable as per DNVGL-ST0164	
		1st torsional	Responses above 100 Hz not recorded			
2	Post-fatigue testing	1st flapwise	7.58	0.132	0.07	
		2nd flapwise	12.11	0.083	Not applicable as per DNVGL-ST0164	
		1st torsional	Responses above 100 Hz not recorded			
3	Post-testing	1st flapwise	7.58	0.132	0.05	
		2nd flapwise	12.11	0.083	Not applicable as per DNVGL-ST0164	
		1st torsional	Responses above 100 Hz not recorded			
4	Post-testing	1st flapwise	13.28	0.075	0.06	Load introduction clamp removed
		2nd flapwise	19.69	0.051	Not applicable as per DNVGL-ST0164	
		1st torsional	Responses above 100 Hz not recorded			

### Static testing

**A. D40 blade**

**Fig. 16:**
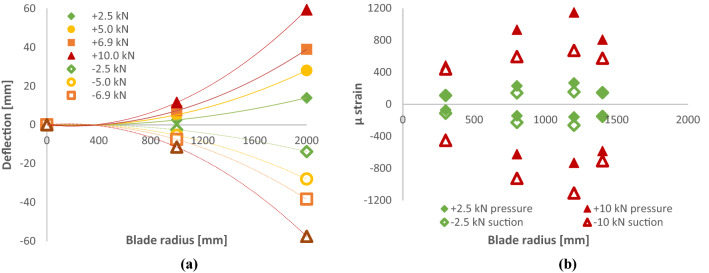
**a** Deflection per blade length up to 10 kN load, **b** Max strains reached during static testing

**Fig. 17:**
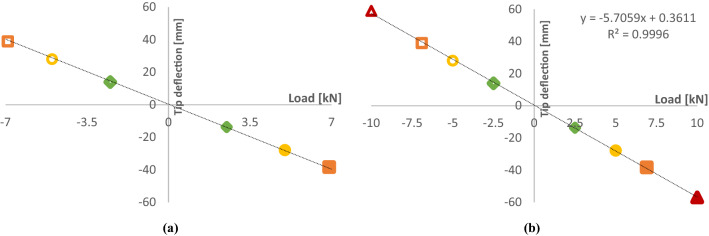
Static tip deflection **a** pre-fatigue testing up to a max of ± 6.9 kN, **b** post-fatigue testing up to ± 10.0 kN

D40 blade tip reached a maximum deflection of + 59 mm under a pressure direction load of + 10 kN. Maximum tip deflection in the suction direction was − 57 mm under a load of − 10 kN. Figure 16 shows this blade deflection at the *r* = 1000 mm station and the tip (*r* = 1950 mm). Figure 17 shows the static tip deflection through the range of loads imparted during the test programme. In Fig. 17, the exceptionally high *R*^2^ value for the linear trendline through the data points indicates that the blade remained in its elastic deformation region throughout the static loading. This is especially significant given that the maximum static load of ± 10.0 kN is 45% greater than the 20-year equivalent fatigue load of ± 6.9 kN.

**B. D63 blade**

**Fig. 18:**
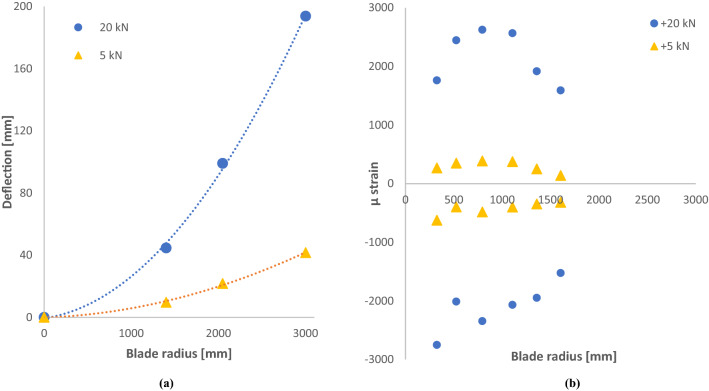
**a** D63 static mid-chord tip displacement, **b** max static strains

Static tests on the D63 blade were also performed in accordance with the IEC and DNV-GL standards (IEC 2020; DNV GL 2015). Tests comprised the pressure direction only, yielding positive loads and displacements. Results of D63 static testing are presented in Fig. 18.

### Fatigue testing

The following sections present the test data gained from fatigue testing of the two blades under investigation. Note that while test data is presented continuously, testing was carried out in discrete blocks of cycles to allow for effective supervision of the test.

**A. D40 blade**

**Fig. 19:**
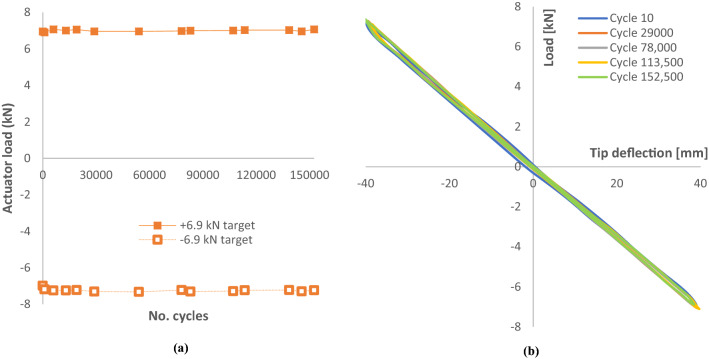
**a** Actuator load at constant displacement during fully reversed fatigue cycling targeting ± 6.9 kN, **b** tip displacement sampled at different cycle numbers throughout the programme

Figure 19a and b show the response of the test blade to displacement-controlled fatigue testing. Load response is seen to change during the initial 10,000 cycles as is normally experienced during ‘bedding-in’ of test infrastructure. Target load during this fatigue programme were ± 6.9 kN fully reversed loading. The tip displacement trace presented in Fig. 19b shows very little change in deflection over the 152,500 cycle programme. Table 9 shows the range of loads reached during 152,500 cycles of fatigue loading at ± 10.0 kN.

**Table 9:** Actual loads and deflections recorded during 152,500 fatigue cycles at ± 6.9 kN

	Pressure direction 6.9 kN target^a^	Suction direction − 6.9 kN target^a^
Max	7.07	− 7.32
Min	6.91	− 6.98
Average	7.00	− 7.23
Range	0.16	0.34
CoV	0.007	0.011

^a^Subject to rounding

**Fig. 20:**
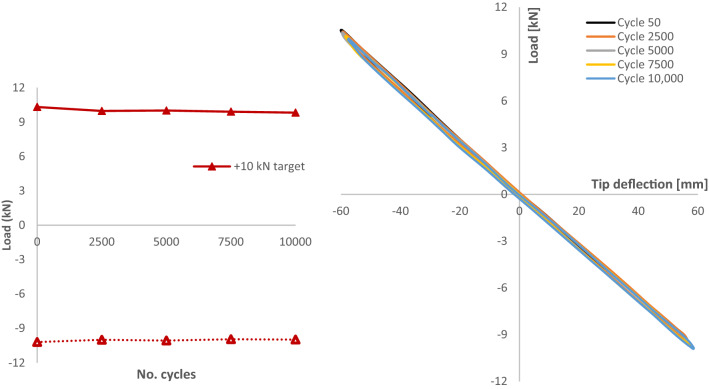
**a** Additional fatigue testing on the D40 blade with target loads of ± 10.0 kN, **b** D40 tip displacement sampled at different cycle numbers throughout the additional fatigue testing

As the test blade passed the main phase of fatigue cycling without any degradation in performance, it was decided to supplement the testing with cycling at higher loads of ± 10.0 kN, up 45% from the design test load of ± 6.9 kN. Similarly, the cycling was displacement controlled and the required displacements were found experimentally through the static testing in Sect. 3.2. Table 10 details the actual loads reached during the additional 10,000 cycles of fatigue loading at ± 10.0 kN (Fig. 20).

**Table 10:** Actual loads and deflections recorded during 10,000 fatigue cycles @ ± 10.0 kN

	Pressure direction + 10.0 kN target^a^	Suction direction − 10.0 kN target^a^
Max	10.3	− 9.94
Min	9.82	− 10.2
Average	10.0	− 10.0
Range	0.48	0.26
CoV	0.019	0.010

^a^Subject to rounding

**B. D63 blade**

**Fig. 21:**
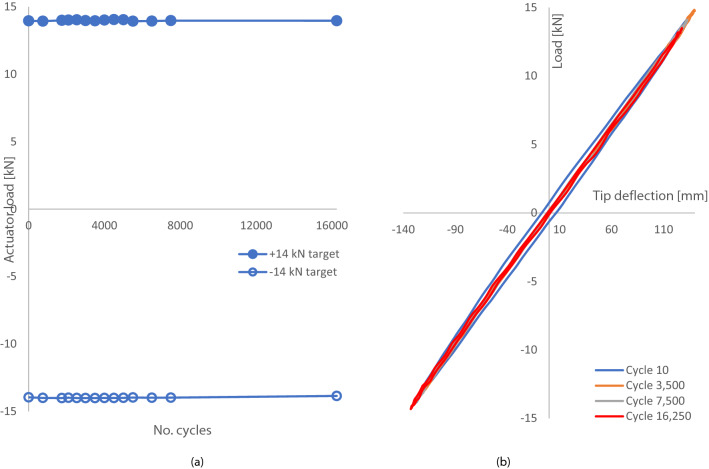
**a** Cyclic testing on the D63 blade with target loads of ± 14.0 kN, **b** D63 tip displacement sampled at different cycle numbers throughout the test programme

Figure 21 shows the load response of the D63 to cyclic loading up to a total of 16,250 cycles at which point the test was discontinued. The load response to the displacement controlled test was very stable throughout the cycles with the load ranges remaining small from test start to finish, as summarised in Table 11.

**Table 11:** Actual loads and deflections recorded during D63 cyclic loading at ± 14.0 kN

	Pressure direction 14.0 kN target*	Suction direction − 14.0 kN target*
Max	14.05	− 14.01
Min	13.94	− 13.85
Average	14.00	− 13.98
Range	0.12	0.16
CoV	0.002	0.002

^a^Subject to rounding

## Discussion

The results presented in this paper show how an effective tidal energy blade can be produced using established manufacturing methods and widely available materials. The benefit this represents for the industry is significant—the tidal energy industry is at a relatively early stage of *technology readiness level* compared to more established forms of renewable energy, so all de-risking activity is welcome to promote confidence in the industry. De-risking, as done in this activity, is particularly helpful as it qualifies already standard practices for use in an emerging application. *Vacuum Assisted Resin Transfer Moulding* (VARTM) is a well-established means of producing composite parts, requiring widely available tools and materials to produce the complex shapes required for efficient energy capture. Likewise, the blades’ passive-adaptive design is attractive to developers as it removes the requirement for complex pitch and load control systems, especially on lower power rated devices where achieving a competitive LCOE is more challenging.

The testing of the D40 and D63 blades also served to test the blade-hub connection. Blade-hub connections are an area of tidal blade manufacture somewhat overlooked, leading to wind-industry derived solutions being leveraged, where this is not the optimum solution. As described in Sect. 2, the continuous fibre approach has been proven to be a suitable candidate for this type of connection. Further development work can now take place in the refinement of this method.

Tidal energy by its nature is site specific and the ability to have tailored devices will be a key factor in meeting Ocean Energy Europe’s 2030 targets as outlined in Sect. 1. This study has succeeded in validating two blade lengths, which were designed and manufactured using the same approach showing the versatility of the methods used by SCHOTTEL Hydro and Sustainable Marine. The output of this study will contribute to the potential for installation on sites that were not previously considered for development due to technology limits.

**Fig. 22:**
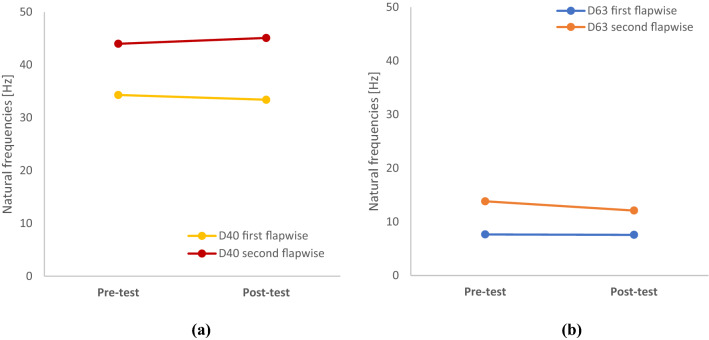
Comparison of flapwise natural frequencies before and after testing, where **a** relates to the D40 blade and **b** to the D63 blade

Natural frequency testing was conducted to determine if there was any relationship between it and blade residual life. Results from the investigation of both blades show no clear change in the blade’s resonant frequencies at different points during the testing. Results of natural frequency testing on both blades are presented in Fig. 22, where they show no pronounced change in blade frequencies after rigorous testing. Natural frequency testing is a straightforward and intuitive type of test to perform on tidal blades as a measure of residual life. However, the apparent lack of relationship between blade damage and the resonant frequency is significant for future developers, as it shows this type of testing may not be a reliable means of quantifying blade damage.

The test programme raised additional discussion on maintaining the validity of test setups at high loads. Given the testing was conducted on more flexible, passive adaptive blades, fatigue testing at the higher load levels was found to be reaching the extremes of rotation capacity of the Shore Western swivel head used to transfer load into the blade. A lesson learned from the testing is that it is important to build in flexibility to the test setup to ensure the blade can be loaded in a manner consistent with what can be expected at the extreme ends of its loading envelope, but also have the capability to be loaded beyond this initial estimate so as to be capable of determining the limit of the blade capacity.

Static testing of the blade yielded valuable deflection and strain data from the significant number of installed sensors, LVDTs and string pots. Blades are often bound in their design by a maximum fibre strain limit and, thus, one of the key outputs of blade design and modelling is an estimate of what this parameter will be during operation. Strain gauges installed at key positions on the blade surface (as outlined in Figs. 16 and 18) recorded both the peak and transient strains during a wide range of static tests. DNVGL-ST-0164 advises that strain limits of ≤ 0.24% in tension and ≥|-0.18%| in compression are ‘sufficiently conservative’ and ‘may therefore be applied without material testing’ when working with carbon-epoxy laminates. Testing of the D63 blade presented above showed the tensile strain to be on the extremities of this.

Blades of the VARTM construction technique with similar fibre type and layup are popular in the area of renewables development; the data from this testing can now be used to inform and validate the future of modelling of similar composite blades. Future tidal blade developers may choose to use the strain data such as that presented in this study to further push the boundaries of what is possible from VARTM-produced carbon blade. Such developments may be:

- Delaying the action of the passive-adaptive load control behaviour to allow the blade operate up to a higher flow speed (i.e. blade loads).
- Extending blade predicted lifetimes based on what was learned in this test programme.
- Push the boundaries of blade design by operating the turbine with higher hydrodynamic loads on blades.

All approaches would lead to further LCOE improvements as more and more energy is gleaned from a given installation.

**Fig. 23:**
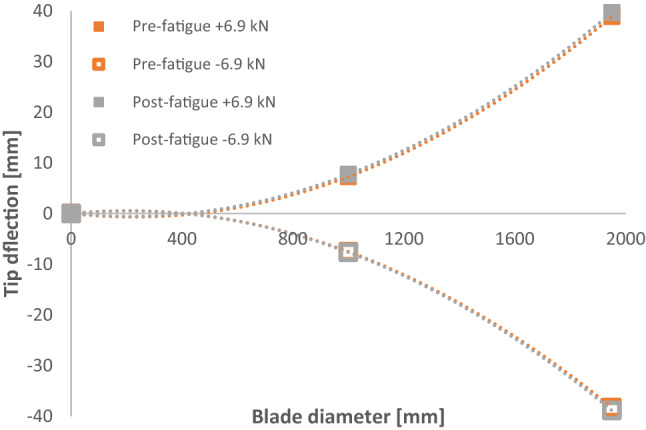
Static displacement of the D40 blade before and after fatigue testing

Fatigue and cyclic testing were key elements of this project which has generated large amounts of data for future tidal turbine blade design and modelling. Testing saw a range of loads imparted to the two blades during the project giving an excellent range of data generated from the testing. Figure 23 shows the effect of the lifetime-equivalent testing on the strength of the D40 blade by comparing the static response of the blade before and after fatigue testing, showing that there has been no significant change in the blade strength due to the fatigue testing programme. The equivalent response of the blade after 152,500 fatigue cycles contributes to the validation of the models used to design the blade.

Lessons learned from cyclic loading of the D63 blade during initial testing generated data that contributed to the lifetime-equivalent testing of the D40 blade.

The longer D63 blade was subjected to a higher cyclic loading of over 200% of the D40 loading. Behaviour of this nature is hugely insightful for future research owing to the data that was generated in a controlled environment where key blade parameters were recorded.

## Conclusion

The purpose of the test programmes was to prove the structural integrity of the SCHOTTEL HYDRO blades over an operational lifetime of 20 years in a laboratory environment using recognised international standards (DNVGL-ST-0164 and IEC-TS-62600–3). To this end, a comprehensive test programme of static and fatigue loading was conducted along with natural frequency testing to trial as a tool for evaluating the residual life of blades. Initial static testing and cyclic testing of the D63 blade were completed. This was followed by initial static testing of the D40 blade up to max loads of ± 6.9 kN which showed a strong linear relationship to tip deflection. Subsequently, a full fatigue testing programme was performed on the D40 blade, where 152,500 cycles were completed, which is the equivalent of a 20-years operation, along with residual strength testing. Therefore, demonstrating both the structural strength and the design life of the SCHOTTEL HYDRO blade.

The net effect of this research is to progress marine energy to becoming a mainstream source of renewable electricity for the European consumer. De-risking of any new technology is key to it gaining investment and traction on the commercial stage, with the citizen being the ultimate beneficiary. Furthermore, the benefits of de-risking marine energy are two-fold—the predictability of tidal energy leads to planning efficiencies in the electrical distribution network which in turn reduces the capacity of the network required. Development work such as this proves that tidal components have the durability to provide a useful life on a par with its counterparts in other renewable technologies, this, in turn, paves the way for certification and removes one of the barriers to commercialisation.

## Data Availability

Publicly available testing data is available in this publication. Additional testing data may be granted through contact with Jamie Goggins.
